# Assessment of the usefulness of presepsin (soluble CD14 subtype) in septic patients

**DOI:** 10.1186/cc10388

**Published:** 2011-10-27

**Authors:** T Nishida, H Ishikura, A Murai, Y Irie, T Umemura, T Kamitani, S Endo

**Affiliations:** 1 Department of Emergency and Critical Care Medicine, Fukuoka University Hospital, Fukuoka, Japan; 2 Department of Critical Care Medicine, School of Medicine, Iwate Medical University, Iwate, Japan

## Introduction

Sepsis is a life-threatening condition characterized by a whole-body inflammatory state. The early diagnosis and treatments of sepsis will improve the outcome of patients. The aim of this study was to compare blood levels of presepsin (renamed from soluble CD14 subtype), procalcitonin (PCT), IL-6 and C-reactive protein (CRP) and to investigate the most useful biomarker for early diagnosis of sepsis.

## Methods

A single-center, prospective, observational study. Patients who had one or more systemic inflammatory response syndrome criteria were included in this study. The blood samples for measuring the biomarkers were collected and the severity of sepsis was evaluated at the time of admission and every other day for a week. Forty-two patients were enrolled for the prospective study from June 2010 to December 2010.

## Results

Twenty-three patients were diagnosed with sepsis and 19 patients were without sepsis. In the receiver operating characteristics (ROC) curve analysis, the area under the curve (AUC) to distinguish sepsis was the largest for presepsin (0.930) followed by IL-6 (0.896), PCT (0.854) and CRP (0.840). Presepsin may be able to discriminate between patient groups with or without sepsis. From the ROC curve analysis, a cut-off value of presepsin was 929 pg/ml with sensitivity and specificity of 76% and 81%, respectively, with odds ratios and 95% CIs of 0.996 (0.992 to 0.998) and 3.376 (1.497 to 6.094). And the presepsin values were significantly higher in the patients with the more severe septic condition (for example, sepsis, severe sepsis, septic shock). In addition, a significant correlation was found between the Sepsis-related Organ Failure Assessment scores and the presepsin values (*r*^2 ^= 0.320; *P *= 0.0003). But there was a no significant correlation between APACHE II scores and the presepsin values.

## Conclusion

In this study, presepsin is the most valuable predictor about sepsis compared with PCT, IL-6 and CRP. Moreover, the results suggest that presepsin values can serve as a parameter that closely reflects the pathology. So we strongly suggest that the presepsin will be not only a very useful new biomarker of the diagnosis of sepsis, but also useful for monitoring the severity of the disease in the near future.

